# A Survey of Practical Design Considerations of Optical Imaging Stabilization Systems for Small Unmanned Aerial Systems

**DOI:** 10.3390/s19214800

**Published:** 2019-11-04

**Authors:** Christopher Dahlin Rodin, Fabio Augusto de Alcantara Andrade, Anthony Reinier Hovenburg, Tor Arne Johansen

**Affiliations:** 1 Department of Engineering Cybernetics, Norwegian University of Science and Technology (NTNU), 7034 Trondheim, Norway; fabio@ieee.org (F.A.d.A.A.); hovenburg@ieee.org (A.R.H.); tor.arne.johansen@ntnu.no (T.A.J.); 2 Drones and Autonomous Systems, NORCE Norwegian Research Centre, 9294 Tromsø, Norway; 3 Graduate Program in Electrical Engineering (PPEEL), Federal Center of Technological Education of Rio de Janeiro (Cefet/RJ), Rio de Janeiro 20271-204, Brazil

**Keywords:** unmanned aerial systems, gimbal stabilization, stabilization systems, dampers, vibration, optical image stabilization, software image stabilization

## Abstract

Optical imaging systems are one of the most common sensors used for collecting data with small Unmanned Aerial Systems (sUAS). Plenty of research exists which present custom-made optical imaging systems for specific missions. However, the research commonly leaves out the explanation of design parameters and considerations taken during the design of the optical imaging system, especially the image stabilization strategy used, which is a significant issue in sUAS imaging missions. This paper surveys useful methodologies for designing a stabilized optical imaging system by presenting an overview of the important aspects that must be addressed in the designing phase and which tools and techniques are available and should be chosen according to the design requirements.

## 1. Introduction

The use of small Unmanned Aerial Systems (sUAS) has increased rapidly during the last years. While some hobby users operate sUAS without any particular purpose, research institutes and corporations commonly operate sUAS for the purpose of collecting information about the environment. Environmental data that can be of interest include readings of the Earth’s magnetic field, elevation data acquired by a LiDAR, and various wavelengths of transmitted and reflected light acquired by a camera system.

Optical imaging systems are one of the most common sensors used for collecting data with sUAS, and their images can be used to take snapshots of beautiful scenery, provide situational awareness to an operator during a mission, and in various photogrammetry applications. Several commercial off-the-shelf optical imaging systems for sUAS exist, in particular for missions with high requirements on the visual appeal of the images rather than the accuracy of the world time, position, and attitude of the camera when the image was acquired. This is often the case for TV and video production, photographers, and hobby users.

Image meta data such as world time, position, and attitude are, however, of high importance in many sUAS missions performed by corporations and research institutes. With the fast development of new sensors and methods to improve the accuracy and decrease the cost of acquiring these meta data, together with a lack of off-the-shelf customizable imaging systems to test the latest technology with, many have created their own payloads. Plenty of research exists which present custom-made optical imaging systems for specific missions [1,2,3,4,5]. The research shows proofs of concept, which can be very valuable since the image acquisition process is highly complex—experimental data from multiple systems can show how design parameters affect the data quality in general, or can be used to replicate the system for a similar mission. However, the research commonly leaves out the explanation of design parameters and considerations taken during the design of the optical imaging system, especially the image stabilization strategy used, which is a significant issue in sUAS imaging missions. On the other side of the spectrum, there is research explaining intricate details of the design of inertially stabilized platforms [6,7].

The present paper aims to contribute with filling this gap in the literature by providing useful guidelines and methodologies for designing a stabilized optical imaging system. However, it does not consider more advanced topics for making a finished product, such as structural analysis, or broad, non-specific topics such as software design. Therefore, the goal of this article is to give an overview of the important aspects that must be addressed when designing a stabilized optical imaging system and which tools and techniques are available and should be chosen according to the design requirements.

This paper is structured as follows. In the beginning of the next section, the main vibration sources and the main techniques used to identify and evaluate them are discussed, as well as their effect on image quality. As there are different ways to administer the vibration issue, the main techniques are presented in the following sections. First, the mechanical installation of dampers is presented, followed by optical image stabilization and software solutions. Finally, the next sections are focused on gimbal design, including the design considerations, followed by the impact of stabilized image systems on the sUAS aerodynamics. Therefore, in the end, the reader can make the appropriate choice of which techniques to be explored, according to the project requirements and limitations.

## 2. Methods

### 2.1. Vibration Sources and Effects

Vibration is one of the main concerns when designing sUAS camera systems: it potentially adds blur, decreasing the image quality and the potential to distinguish detail in the image, and consequently has the potential to compromise the entire mission. Therefore, it is important that the causes of vibration are understood so that the proper mitigation actions are taken.

#### 2.1.1. Vibration Sources in sUAS Platforms

This section goes through the main sources of vibration in the two main popular designs of sUAS platforms: fixed-wing and rotary-wing small Unmanned Aerial Systems. Some sources are shared between them, such as rotors/propellers, and other sources are fairly unique to each platform (e.g., combustion engine of fixed-wing sUAS).

##### Fixed-Wing Platforms

In [8], with the objective to choose the most suitable sUAS for LiDAR mapping, the authors studied the vibration, capacity, reliability and stability of many sUAS platforms. With the acquired knowledge about the different platforms, they developed two sUAS especially optimized for the LiDAR mapping. With respect to the sources of vibration on fixed-wing sUAS, the authors based their theoretical analysis on a study performed by Ma and Wu [9] about positioning errors on LiDAR systems caused by manned aircrafts platform vibration. In this study, the author enumerated four main sources of vibration on manned fixed-wing aircrafts: engine, external wind flow, internal wind flow within open cavities, and airframe structural motions. Regarding the combustion engine, its noise impinges on the aircraft structures, causing vibrations mainly on the frequency of the engine’s rotation speed and also on double frequency, from the reciprocating motion of the piston. The second and third main sources of vibration are both due to turbulent aerodynamic flow. One is caused by the flow over external aircraft structures and the other is caused by the flow and acoustic resonance phenomena within cavities open to the external airflow. However, according to Uragun and Tansel [10], these vibrations can be considered less significant for sUAS due to lower speeds compare to the commercial and military aircraft. Finally, the fourth main source of vibration pointed by Ma and Wu [9] is with regards to airframe structural motion caused by maneuvers, aerodynamic buffet, landing, taxi, etc. Vibration can also be also caused by specific installed items, however, according to the authors, the effect is only locally on the surroundings of the item.

##### Rotary-Wing Platforms

Battery powered rotary-wing sUAS have the rotors as the main source of vibration [11,12]. In [12], measurements were performed for three setups: motor without propeller, plastic propeller, and wooden propeller. For the first setup, where no propeller was mounted, low force levels for both radial and axial vibrations were recorded, indicating that propellers are the main sources of vibration. In that case, the frequency of vibration is related to the rotation speed of the rotors/propellers. According to Zhenming Li et al. [11], the second main source of vibration on rotary-wing sUAS corresponds to the vibration of the sUAS structure, mainly the platform and extension arms. However, the frequencies of structural natural frequency vibrations depend on the sUAS structure. Verbeke and Debruyne [12] compared numerical simulations of the vibrations on a hexacopter structure using finite elements (FE) model with experimental results obtained by the impulse hammer excitation method. The results were satisfactory, achieving a vibration frequency accuracy between 0.047% and 2.852%. In addition, a third source of vibration was identified by Zhenming Li et al. [11], caused by the vibration of the payload, such as batteries and other weight sources located on the bottom of the sUAS.

#### 2.1.2. Vibration Effects on sUAS Image Quality

**Figure 1:**
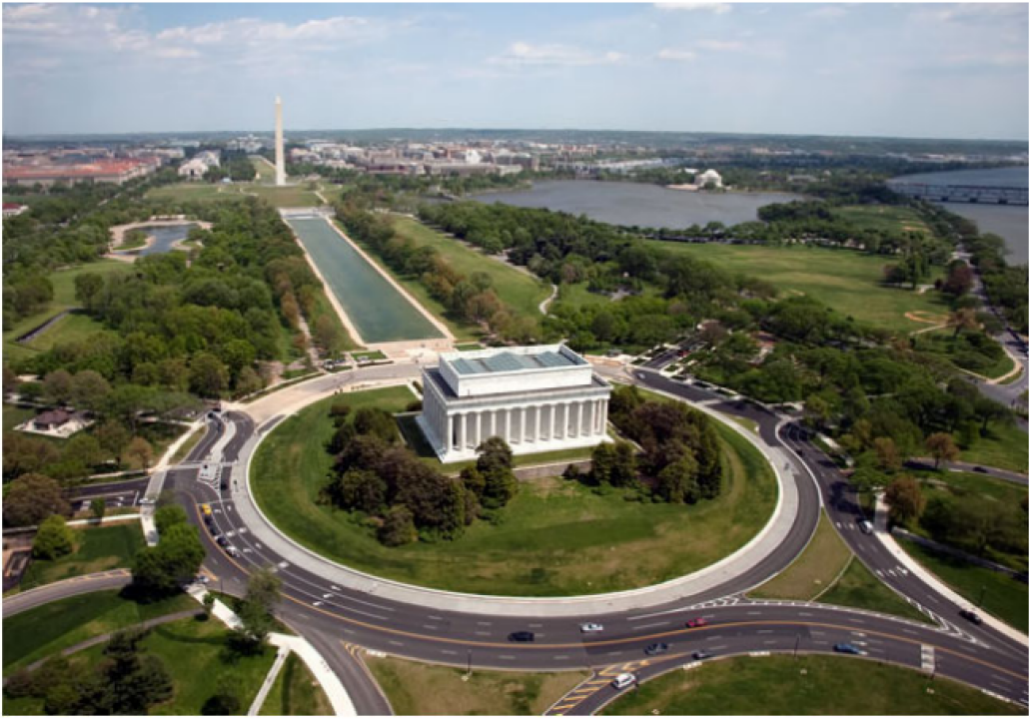
Original image [13].

**Figure 2:**
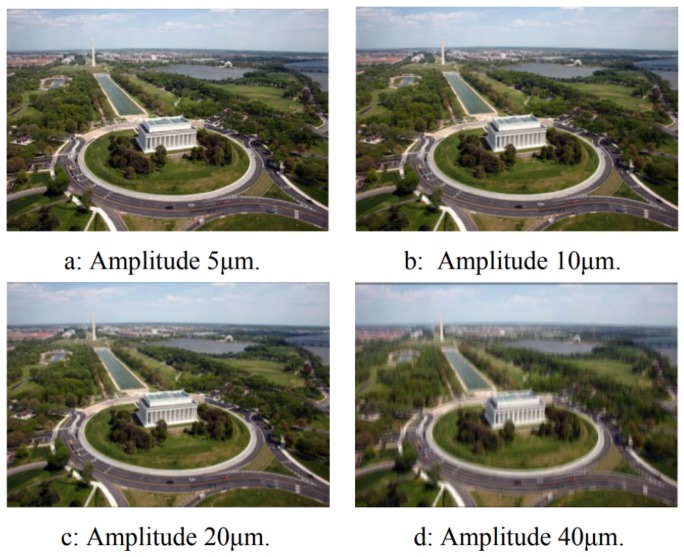
Vibration simulations with different amplitudes [13].

There is not a significant number of studies about the effect of sUAS vibration on image quality. Li and Tan [13] investigated the effects of sUAS vibration on Binary Optical Elements (BOE). BOE is a diffraction imaging element and the diffraction efficiency can be impinged by the vibration of the platform, affecting the image quality. In other words, the relative position between the object point and the optical system changes by the movement of the platform, deteriorating the quality of the image. Therefore, the study simulates the effect of one dimension sinusoidal vibrations with different amplitudes and frequencies on an image with pixel size of 9 μm and integration time of 20 ms (Figure 1). First, different amplitudes at a constant frequency of 50 Hz are applied (Figure 2). For 5 μm of amplitude, no significant changes on the quality can be noticed. For 10 μm of amplitude, the image quality is affected; however, a more significant degradation is noticed on the edges of the image than in its center. For 20 μm of amplitude, the whole image gets blurred, and, for 40 μm of amplitude, the image quality is heavily affected.

**Figure 3:**
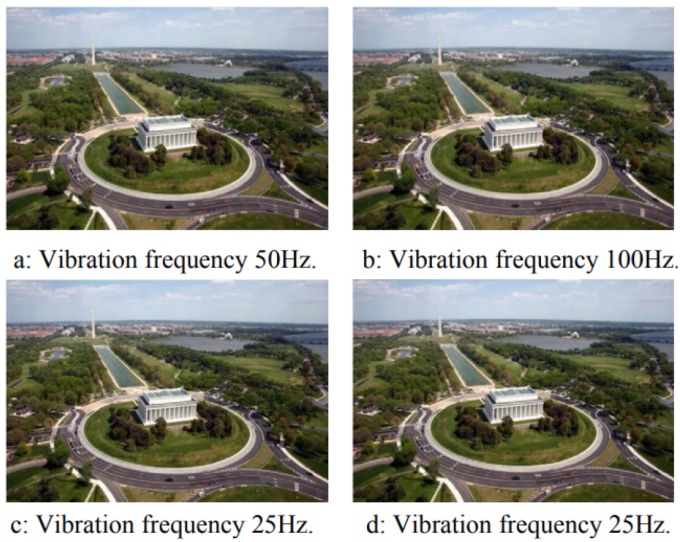
Simulations with different frequencies [13].

Figure 3 shows the result when the same vibration amplitude (15 μm) is applied to the image, but with different frequencies. There are no significant differences between the images in Figure 3a,b. The conclusion is that, when a vibration period is lower than the integration time, increasing the vibration frequency has little effect on the image quality. The images in Figure 3c,d have the same frequency and amplitude, but the vibration was applied on different moments. This may be due to the varying speed of the camera during sinusoidal vibration. This means that, in the case of low-frequency vibrations, the resulting image quality can be different for different time periods since the phase between the vibrations and the image integration period is likely to shift randomly during a flight.

Based on the results, Li and Tan [13] proposed two modifications on the optical system to reduce the effect of vibration on the image quality. As small amplitudes of vibration cause minor effects, one way to reduce the degradation of image quality is to reduce the focal length and increase the pixel size. However, this will result in a decrease in the angular resolution of the optical system. Another way is to use a CCD (charge-coupled device) with a shorter integration time, so that the vibration period is lower than the integration time.

**Figure 4:**
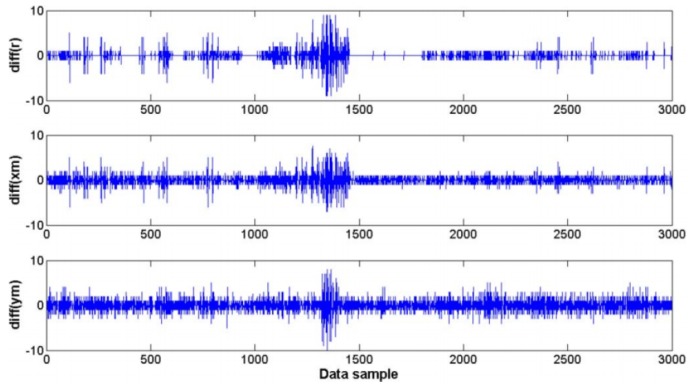
Estimated displacement of the center of the fish-eye image in pixels (xm, ym) and the estimated radius of the entire fish-eye lens image circle boundary (r) [15].

**Figure 5:**
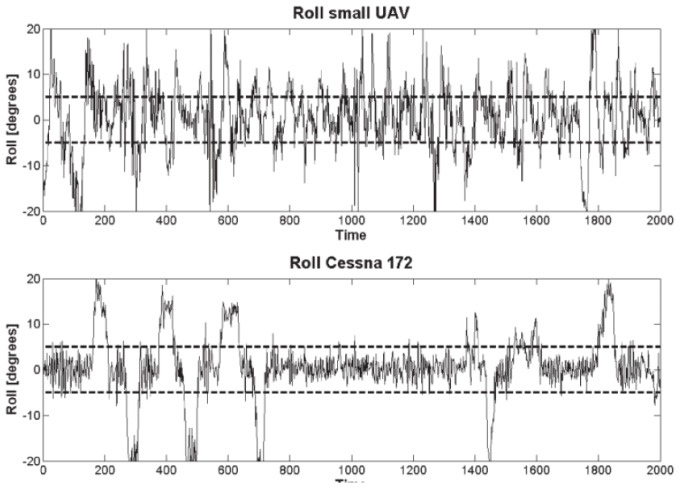
Comparison of roll angles on sUAS and manned aircraft [15].

Other studies that investigated the effect of sUAS vibration on image quality were performed by the authors of [14,15]. The latter study not only focused on vibration but also investigated other aspects such as lens calibration, orthorectification and mapping. Both studies worked with images taken on the same research mission and by a Point Grey Research “Flea” camera with a Fujinon YV 2.2 × 1.4 A-2 fish-eye lens mounted both on a sUAS and on a manned aircraft (Cessna 172). In the sUAS setup, the camera was mounted on a small supporting platform that was isolated from vibrations using a special anti-shock material. In the manned aircraft, the camera was mounted on a simple mounting bracket. The main problem caused by the aircraft vibration was the movement of the lens relative position to the camera (Figure 4). The authors concluded that the non-interference fit between the camera and the lens housing is responsible for the vibration effect. In addition, the problem of vibration is more noticeable on the sUAS, because of its small size, making it more susceptible to maneuvers and turbulence. The comparison between the roll angle noise of the sUAS and the manned aircraft can be seen in Figure 5. The sUAS has variations approximately twice of the variations on the Cessna 172. In [14], where the focus of the investigation was on vibration effects and compensation, the vibration was divided into rotation and translational vibrations. The largest translational movement detected was ±5 pixels measured from randomly selected images. That is a big issue since vibrations causing only a one-pixel shift in a fisheye image captured by a sUAS operating at an altitude of 1000 ft above the ground would result in a displacement of approximately 2.5 m, using a 0.8 megapixel resolution camera.

The available literature about sUAS vibration and image quality is very limited. However, there are a few studies about the effect of mechanical platform vibration on satellite imaging (e.g., [16]). In this case, vibrations limit the maximum resolution and performance of remote sensing and are caused by turbines, motors, reaction wheels, actuators, etc. The study performed by Haghshenas [16] was based on the work of Wulich and Kopeika [17], who analyzed the relation between blur, vibration, exposure time and resolution, with focus on vehicular or airborne imaging systems and in robotic systems. The calculations can be used to determine the most appropriate sensor for a given task, and the number of images of the same scene that are necessary to achieve a required resolution.

### 2.2. Mechanical Vibration Mitigation

Dampers are very popular devices for vibration mitigation. A wide selection of vibration dampers are available off-the-shelf. The knowledge of how to select the best damper is however not widespread, and it is common to just try a few and see which one works best. By using a more systematic design method together with collected vibration data (or estimated from the vibration sources), it is possible to remove targeted vibration frequencies more efficiently. Since trial-and-error might be resource-intensive in both man-hours and components, a systematic approach is likely to reduce both cost and time of developing a stabilizing system.

In addition, to reduce the vibration effects, actions can be taken in the sUAS platform design phase, when selecting the materials and when designing the structure.

#### 2.2.1. Dampers on the Optical Imaging System

**Figure 6:**
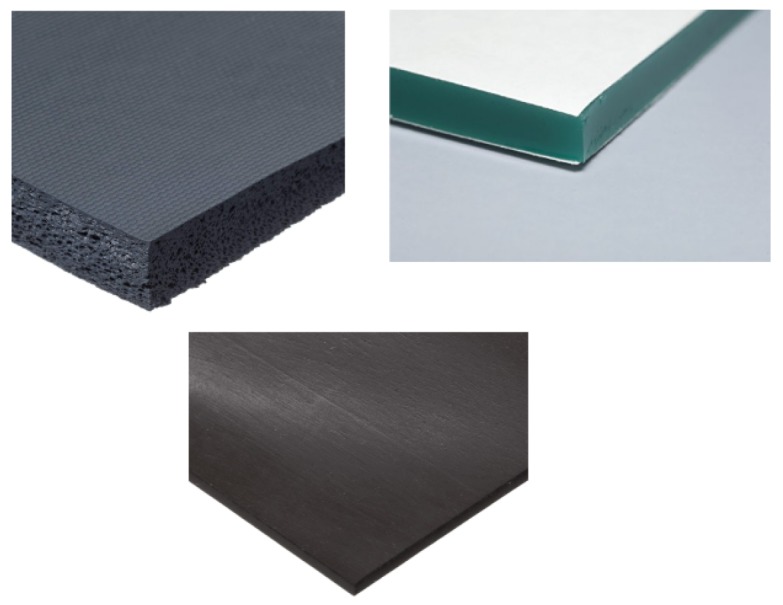
Silicone Foam (**top left**); Kyosho Zeal (**top right**); and Durometer Sheet (**bottom**) (adapted from Amazon.com).

Dampers can be used for vibration isolation, to lower the natural frequency of the system to below the excitation frequency, and for vibration damping, where the aim is to absorb the mechanical energy and convert it to other energy forms, such as heat. Three types of dampers (Silicone Foam; Kyosho Zeal; and Sorbothane 30 Durometer Sheets) (Figure 6) were tested by Zhenming Li et al. [11] to mitigate the vibration on a rotary-wing sUAS. In total, six aspects were taken into consideration when choosing the dampers: (1) electrical insulator to avoid short-circuit; (2) soft and flexible; (3) natural frequency outside sUAS structural resonance zone; (4) low compression set and low creep; (5) good resistance to outdoor conditions; and (6) easy installation and adjustment.

**Figure 7:**
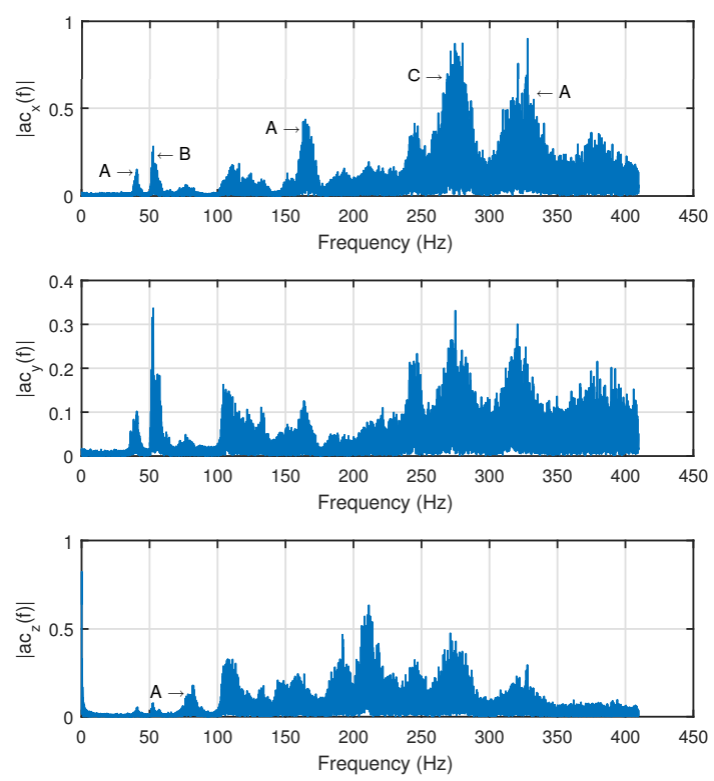
sUAS vibration measured by an IMU before the damper installation [11].

To study the effect of the dampers on the sUAS vibration mitigation, a structural vibration analysis was done before the installation of the dampers. In the first step, the sUAS structure was modeled in SolidWorks simulation, considering also the materials properties. The frequency analysis was carried out and the high vibration frequencies observed were 39.90 Hz on the x- and y-axes, and 80.48 Hz on the z-axis. Similar behavior also existed for 160.17 Hz and 321.82 Hz, respectively, which the author suspected were the third- and fourth-mode natural frequencies, related to the payload. Small vibrations were obtained between 100 and 200 Hz and a stronger one at 273.16 Hz. The last ones were probably related to the structure, such as arm extensions and components. After the simulation, flight tests were performed and the vibrations were measured by an additional IMU with a sampling rate above 800 Hz. The results (Figure 7) show that the vibration data obtained on the flight tests was very similar to the simulated one. Peaks (A) related to the payload were verified on the vibration frequencies of 40, 80, 160 and 320 Hz, a peak (C) related to the structure was verified on around 270 Hz, and another peak (B) was verified on around 50 Hz, which is related to the rotation of the rotors. The last one was not verified in the simulations because the vibration related to the rotation of the rotors was not included on the simulated model.

**Figure 8:**
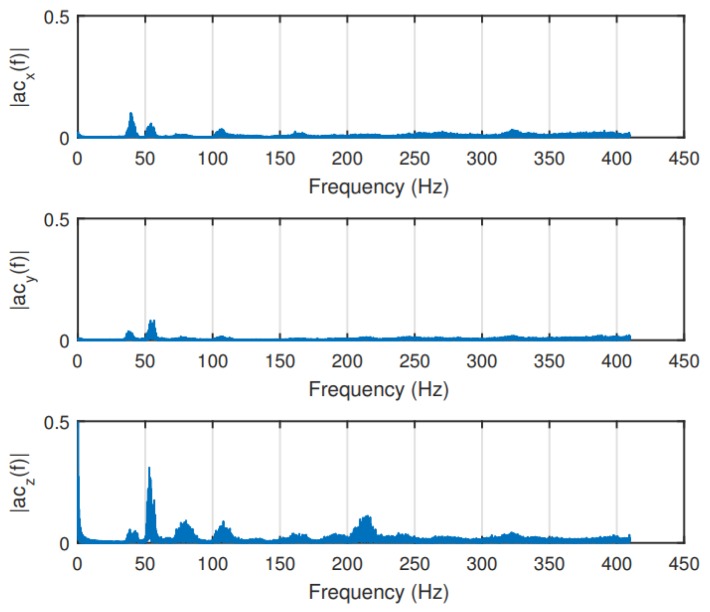
sUAS vibration measured by an IMU after the damper installation [11].

The tests of the effectivity of the dampers were performed on a lab vibration table for a frequency range from 10 to 300 Hz. Different sizes of each of the three dampers were used in order to change the transmissibility curve. Among the selections, Kyosho Zeal Sheet had the best performance. Therefore, it was installed on the sUAS to mitigate the vibrations on the additional IMU. The size of the damper was chosen so that the natural frequency of the damper (around 50 Hz) was as far apart as possible from the highest disturbing frequencies (around 270 Hz) to be mitigated. The results of the flight experiment (Figure 8) show a significant reduction on the vibrations. However, as expected from the transmissibility curve of the damper, vibration caused by the rotors at around 50 Hz was slightly amplified.

#### 2.2.2. Other Mechanical Solutions

In addition to the use of dampers on the optical imaging system, other actions can be taken to reduce vibrations on sUAS. In rotary-wing platforms, an accurate balancing of the propeller blades may reduce the propeller-induced vibrations significantly [18]. Other suggestions were given by Uragun and Tansel [10], focusing on the reduction of noise produced by sUAS, but, as in many cases the noise is related to the vibration of parts of the platform, the same actions can be applied to vibration mitigation. The author categorized the methods into five groups: conventional noise control methods by modifying the structure, futuristic methods, reduction of engine noise, operation time adjustment-based approach, and noise reduction targets of the federal agencies. The first method consists of passive and active techniques. An example of a passive technique is to use vibration-absorbing materials on the structure and therefore reduce the vibration on a specific frequency band. Active techniques are, e.g., closed loop adaptive feed forward control techniques with electromechanical systems, such as piezoelectric actuators, to reduce the vibration of surfaces [19]. The second category is regarding the design and selection of materials that could reduce the vibration, such as “owl wings”. Engine noise mitigation is the third category and the author pointed out some methods from the literature, such as structural modifications, active noise control systems, slightly changing the phase between the propeller sets, and modifying blades and controlling the rotation speed. All these techniques are to be considered during the design and manufacturing phases and should be taken into account if the purpose of the sUAS being developed is sensitive to vibrations. The last two categories discussed by the author are not related to vibration mitigation.

##### Flow-Induced Oscillations

During the design of an optical imaging system, it may prove beneficial to consider the effects of flow induced oscillations on the structure. By minimizing the occurrence of the oscillations, the overall system performance may increase, while the need for mitigation through dampening could be reduced. Here, the most relevant aspects with regards to the stabilized imaging system design are discussed. Vortex-induced vibrations (VIV) is a phenomenon where the generated vortices cause vibrations of the object. This is caused by the asynchronous periodical release of vortices along the object. The magnitude and impact of VIV is highly dependent on the flow conditions and the shape of the body.

Because the optical imaging sensor is mounted on the airframe, it may experience vibrations that are not necessarily caused by the optical imaging sensor itself, but by the airframe or components that are installed on the airframe. Besides more obvious sources of vibrations, the aircraft may suffer from secondary aerodynamic effects. One prominent example of this is *Dutch roll*. This is an oscillatory movement caused by a change in the aircraft’s yaw, which is coupled into a roll movement. Because a yaw movement forces one wing forward in relation to the other, a differential in that wing’s lift and drag occurs. This causes the aircraft to wiggle. The dynamics behind Dutch roll are considered a difficult dynamic mode to analyze [20]. However, if the performance of the optical imaging sensor suffers from the effects of Dutch roll, the typical remedies include an increase of the aircraft’s vertical tail, or the installation of a yaw dampener [21]. The occurrence of Dutch roll can be recognized by an oscillatory motion, where the roll motion lags behind the yaw motion by approximately π/2.

### 2.3. Optical Image Stabilization

**Figure 9:**
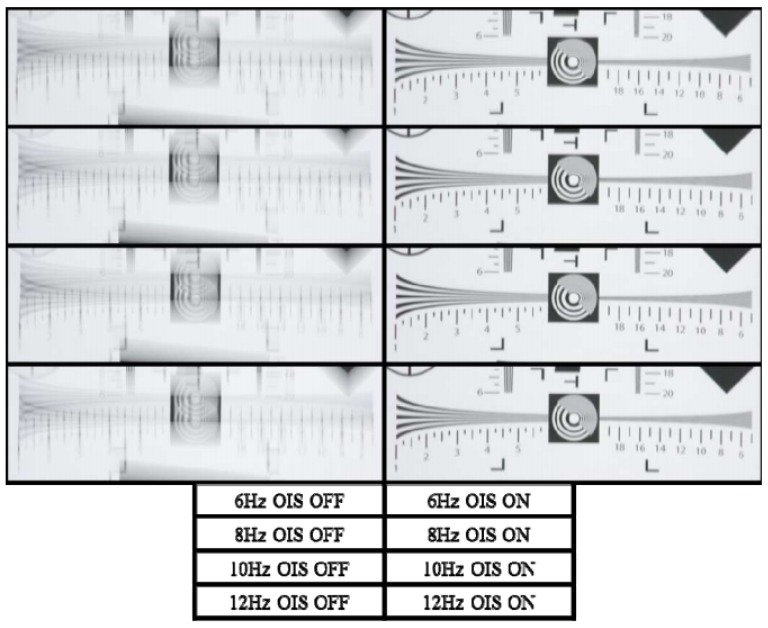
Comparison of OIS OFF and ON on a standard ISO-12233 chart [23].

In imaging missions, an alternative or supplement to mechanical vibration mitigation is Optical Image Stabilization (OIS) [22]. This technique consists of using motion sensors readings to detect vibration and to move the lens or sensor in order to correct the jitter. Nowadays, many camera systems, especially the ones installed on the most modern phones come with this capability. Basically, actuators move the camera system parts according to the detected vibrations, cancelling the effect. OIS is considered superior to digital image stabilization as it acts before the image acquisition and therefore there is no image distortion or degradation. Despite its advantage, not all imaging systems have this feature and the installation of OIS on the existing imaging systems is very challenging as the intervention happens in the hardware of the imaging system. OIS can also alter camera parameters, reducing the accuracy of remote sensing data. Li et al. [23] evaluated the performance of an OIS system using fuzzy sliding-mode controller under the effect of sinusoidal signals of 6, 8, 10, and 12 Hz. The camera acquired images of a standard ISO-12233 chart with OIS ON and OFF. Figure 9 shows the comparison between the chart picture taken from a camera with and without OIS turned on for vibrations of ±0.15 degrees on the vertical axis.

### 2.4. Software Image Stabilization

Image stabilization algorithms are also a way to reduce the effect of vibration on images. This category of image processing is usually referred to as Digital Image Stabilization (DIS). It is important to make a distinction between DIS and digital video stabilization. Digital video stabilization consists of removing the effects of unwanted camera motion from video data; and Digital Image Stabilization (DIS) consists of correcting the effects of unwanted motions that are taking place during the integration time of a single image or video frame [24], by estimating the motion between frames in sequential imaging and then removing unwanted camera motions.

#### 2.4.1. Digital Image Stabilization

In DIS, motion estimation techniques can be classified into two categories: feature-based [25] or direct pixels-based [26] (also called “image-based”). The main difference is that feature-based approaches extract characteristics of the frames such as corners, edges, etc., while direct pixels-based approaches use every single pixel on the calculations. Therefore, techniques using the feature-based approach are usually faster and more effective but implies non optimal use of the available information. In addition, in images where the degradation caused by vibrations is too accentuated, the number of detectable features is small and the features may not be sufficiently reliable, therefore, a direct pixels-based approach would be more suitable because it uses the intensity of every single pixel of the image. The motion estimation is usually done by estimating a parameter vector, which is a two-dimensional mapping function that overlaps input images over a reference image [24]. The reference image has to be chosen among a sequence of images and a good candidate to be a reference image may be the one that is the least affected by blur. To identify such image, a sharpness measure can be used.

**Figure 10:**
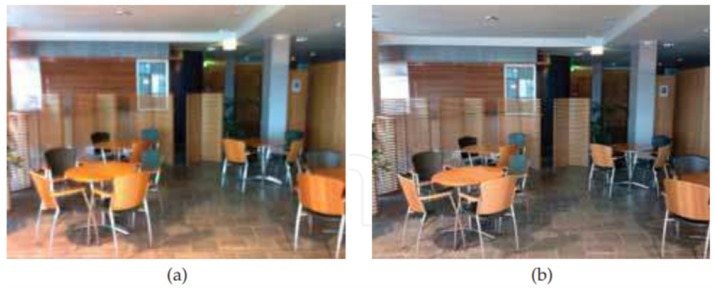
(**a**) Non-stabilized image taken with exposure time of 1.8 s; and (**b**) stabilized image by fusing four frames with exposure time of 0.3 s each [24].

Figure 10 shows an example of a comparison between an image captured with exposure time of 1.8 s where DIS was not applied (Figure 10a) and a resulted DIS image (Figure 10b) using four frames captured with 0.3 s of exposure time each. It is possible to notice that the image for which the DIS algorithm was applied is less blurry.

#### 2.4.2. Digital Video Stabilization

In digital video stabilization, where the goal is to make the video flow less trembled due to the movement of the camera, the motion between frames is also estimated by calculating the rotation and translation between frames. Then, the opposite motion can be applied to counteract image shake and realign the frames in order to make the transition between frames smoother [27]. This is a very popular topic on sUAS imaging because video taken from sUAS frequently suffers from unwanted motion of the sensors.

In [28], Scale Invariant Feature transform (SIFT) was used for key point detection and matching between successive frames taken by a sUAS. Then, an affine transformation model was used to estimate the global motion parameters between two successive frames. After that, the undesired motions were compensated and spatiotemporal filtering was used to remove the noises in the video. Finally, all frames were transformed to obtain stabilized video frames.

**Figure 11:**
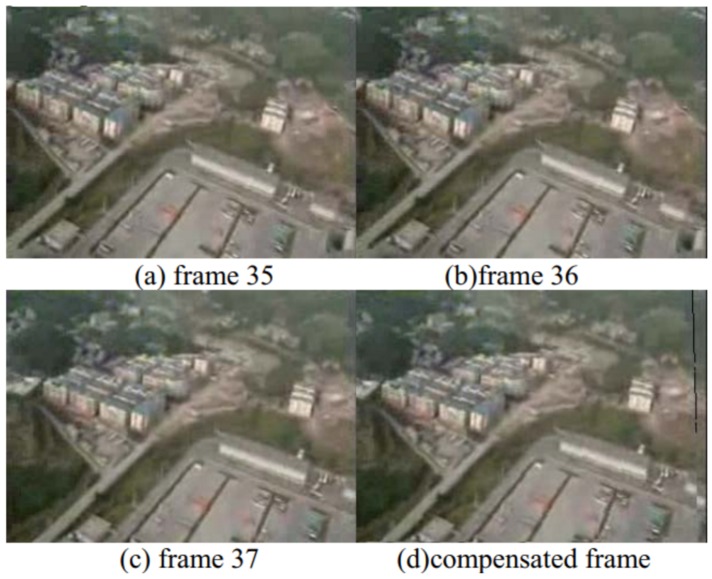
Compensation of an undesired frame for video stabilization [29].

A fast video stabilization for sUAS was proposed by Shen et al. [29]. A polynomial fitting and predicting method was proposed to estimate the global motion parameters and to select undesired frames. After that, the undesired frames are compensated and all frames are transformed to obtain stabilized images. Figure 11 shows the compensation (Figure 11d) of an undesired frame (Figure 11c).

As most of sUAS are equipped with inertial measurement units (IMU), an alternative to the feature- or pixel-based motion estimation for digital video stabilization is to use the IMU readings to calculate the camera motion between frames and use this information to stabilize the camera feed [30]. IMU readings can also be integrated with conventional motion estimation methods to increase the speed and accuracy [31]. In this case, the results can be improved significantly as the accuracy of the motion estimation increases. A timing and navigation solution was developed by Albrektsen and Johansen [32], which made it possible to synchronize the camera images with the sUAS position with high accuracy by using dedicate hardware time synchronization of GNSS, IMU and camera sensors readings.

### 2.5. Gimbal Stabilization

sUAS gimbal systems are electromechanical devices that can be used to stabilize a platform on a given attitude. Therefore, they are suitable to mitigate unwanted camera rotations caused by the UAS motion and also for pointing the platform on a desired direction, controlling the sensor’s line of sight (LoS). Such systems have already been used in many areas before, such as spacecrafts, manned aviation and cinematography. However, due to the recent availability of small UAS and its market growth, there is a new demand for small and precise gimbal systems specifically optimized for particular requirements regarding size and precision. In addition, this topic is benefiting from the advances in the miniaturization of key technologies such as high-performance gyros and drivetrain components, fast embedded microcontrollers and small cameras. Therefore, small gimbal system design is a topic just recently being researched, the main challenges of which are regarding the limited size and weight of the device. To evaluate the different gimbal designs, Miller et al. [33] undertook a number of trade studies, investigating various gimbal configurations, sensors, encoders, drivetrain configurations, control system techniques, packaging, etc.

#### 2.5.1. Gimbal Systems Classification

Miller et al. [33] classified the gimbal systems according to stabilization performance related to the LoS Jitter (μrad RMS). Low performance gimbal systems are the ones with more than 250 μrad RMS of LoS Jitter. Medium quality with from 25 to 250 μrad RMS and high quality with less than 25 μrad RMS. Brake [34] classified the gimbal systems by crossing size and LoS stabilization performance in degrees. Small gimbal systems weight up to 4.5 kg and can achieve a LoS stabilization performance on the order of ±0.5 to ±0.1 degrees. Medium and larger gimbal systems weigh from 4.5 to 9.0 kg and greater than 22.5 kg can and can achieve less than ±0.1 degrees of LoS stabilization performance.

#### 2.5.2. Gimbal Systems Design Considerations

Regarding sUAS gimbal systems design, while Miller et al. [33] did a wide comparison between different configurations to provide a broad overview of the design concepts, Brake [34] focused his work on studying the topic to define the best approach for the development of his specific gimbal system, designed to meet size, weight and performance requirements previously defined.

According to Miller et al. [33], the first aspect of gimbal system design is to decide the number of gimbal axes needed for a desired LoS control and field-of-regard (FoR), which is the area over which the gimbal can point. A minimum of two axes are required for controlling two degrees of freedom and point the LoS in a two-dimensional (vertical/tilt/pitch and horizontal/pan/yaw) desired direction. To control a third degree of freedom, such as the image orientation, a third axis is needed. In small sUAS, two-axis gimbal systems are most commonly used. In fixed-wing sUAS, it is common to use gimbal systems with an outer azimuth gimbal axis to control the pan so that it is able to rotate 360 degrees and therefore have a wider FoR.

Another important design aspect is to correctly align the center of gravity with the gimbal motors. By doing this, the required torque and power to make precise angular rotations can be greatly reduced [35].

Thermal considerations must also be addressed because sUAS gimbal systems are commonly too small to package cooling fans or heat exchangers. Therefore, correct electronics layout and proper materials selection are the best measures to mitigate thermal problems.

#### 2.5.3. Stabilization

As the gimbal system will be mounted on a sUAS, which is subject to vibrations caused by rotors, engine or turbulent aerodynamic flows, the gimbal system vibration isolation must be addressed. Combustion engine powered sUAS produce large torque pulses, due to the non-continuous nature of their operation, often in the range of 50–80 Hz. This can cause significant image blurring and/or excitation of jitter in the gimbal’s control system, if no specific vibration isolation is provided. Electric powered sUAS produce higher frequencies, which are easier to mitigate and have less effect on the image quality.

Gimbal system stabilization can be active or passive. Passive stabilization is related to the fact that the platform, sensor, and target LoS move within inertial space. Therefore, low friction joints and high inner axis inertia can passively contribute to maintaining the desired LoS/attitude [34]. Active stabilization is done when the drivers will act based on sensor readings to keep the platform’s desired attitude/LoS. Therefore, to mitigate these vibration effects, Brake [34] designed an active inertial dampening to take care of frequencies of less than 5 Hz and the gimbal system mechanical design provides a good passive inertial dampening for frequencies on the order of 5–20 Hz. For higher frequencies, the gimbal mounting system is responsible for dampening them out. According to Miller et al. [33], an option to mitigate severe effects of sUAS vibration could be to decouple the gimbal system from the sUAS by vibration isolation, isolating its parts from the sUAS structure; however, this solution may degrade the accuracy of any type of pointing relative to the vehicle, and can induce angular motion inputs. This degradation occurs due to the necessity of accurate measurements the gimbal position relative to the sUAS in order to send the correct controls to the drive systems.

Regarding the vibration caused by the gimbal system structure itself, fortunately, the frequency of the gimbal system structural resonance is typically higher for smaller gimbals than larger gimbals [33]. Therefore, structural vibration effects are often much less a design issue in small sUAS gimbal system design but nonetheless important to consider. The most common approach to deal with this is to include structural notch filters, which helps to improve the loop gain margin at the resonant frequency. Hilkert [6] performed a deeper analysis of the gimbal structural interactions and suggested stiffening the structure, as a first attempt to attenuate LoS motion due to bending and to modify the relevant structural transfer functions. The author also suggested stiffening the torsional response of the mounting structure, adding mass to the stationary gimbal structure, and employing the notch filters in the pointing servo system to achieve a better interaction of the control system with the structure.

##### Drive System

The gimbal drive system can be direct, where the motor controls the axis directly, or indirect, via cables, gears or belts. Gimbals with brushless DC direct drive have the highest performance, being able to achieve very low friction and no reflected inertia [33]. However, it is usually heavier, bigger and more expensive than the other approaches to achieve the same torque, and needs more complex electronics. If an indirect drive with gears or belts is chosen, the solution is cheaper and smaller, but has increased backlash, hysteresis, cogging and compliance as result. The cable drive approach has a performance between the direct drive and gears/belts approaches but it has higher friction and lower stiffness, and difficulty achieving 360 degree continuous motion for the yaw axis. Brake [34] designed the gimbal system using brushless DC servomotors with belts, pulleys and gears for its axes. The first design attempt achieved too high backslash in the pan axis. Therefore, the final decision was to use the motor without a gearbox driving a small rubber wheel directly on an interior bearing surface.

As part of a fixed-wing sUAS imaging system design, Skjong and Nundal [36] designed and produced a new gimbal system aiming to achieve a better stabilization, wire handling, repairing capability and robustness than the off-the-shelf gimbal systems. As self stabilizing direct drive gimbals using speedy brushless motors became available in the market for multi-rotors, the authors decided to use this kind of motor on their new gimbal system design because they allow gimbals to move fast enough to stabilize the camera from low frequency vibrations. The commercial brushless gimbal systems are often supplied with a dedicated controller which uses input from an IMU to be mounted on the camera. However, according to the authors, angular drift in heading is a potential worry when using a brushless motor for the yaw rotation, especially because of the bias instability of the IMU. A possible solution to remediate this problem could be to implement an estimator between the IMU and the controller board, integrated to the sUAS’ heading estimator. Another advantage with using brushless motors and IMU is no need to index the gears when disassembling the gimbal. Therefore, as long as the IMU is reinstalled in the same location and orientation, the gimbal will calibrate itself on start-up.

The minimum torque of a motor used to stabilize a given sensor is the sensor’s moment of inertia times the desired angular acceleration.

(1) T=Jα,

where *T* is the torque, *J* is the moment of inertia and *α* is the angular acceleration.

Therefore, the first steps when choosing the right motor is to calculate the moment of inertia and to choose the desired angular acceleration. Brake [34] derived the equations detailed by Kennedy and Kennedy [37], which include all torque contributions and consequences. In the same axis, the contributions are from the torque of friction and cable restraint and the mass imbalance torque. In the case of the inner axis (e.g., elevation), where the gimbal is mounted on the sensor body, the mass imbalance torque is caused by the asymmetry of the sensor. In the outer axis (e.g., azimuth), where the gimbal is mounted on the inner gimbal mounting that is connected to the sensor body, the mass imbalance torque is caused by the asymmetry of the sensor plus the inner gimbal mounting asymmetry.

In [35], where a gimbal system to house two imaging sensors was designed, the authors also opted to use brushless DC motors to directly drive the gimbal axes because of their superior small angular rotations compared to servos. Low weight motors (109 g per motor) able to carry the payload (around 400 g) were chosen.

##### Motion Sensors

Gyros are the main rotational sensors used in gimbal systems. They measure gimbal angular velocities and are used as system’s feedback. Gimbal gyros are usually based on Micro-Electro-Mechanical Systems (MEMS) technology and should have the lowest noise and bias instability that the design constraints allow.

Resolvers and encoders are the most common gimbal angle transducers [33]. They are used to detect the orientation and to report the absolute position of the axes. Resolvers are more robust but incremental encoders are becoming very popular for gimbal angle measurement because they are smaller, lighter and cheaper and can achieve comparable resolution and accuracy. Encoders can be optical, capacitive or magnetic. Brake [34] used a 12 bits of resolution magnetic encoder for each axis of the gimbal system design.

### 2.6. Impact of the Stabilized Imaging System on sUAS Aerodynamics

Implementing a stabilized imaging system that is optimized for airborne vehicles requires consideration of its effects on the in-flight performance. Studying the effects early in the design process may enable a reduction in the negative impact on the in-flight performance. Presented in this section are the system design trade-offs in relation to the overall aircraft performance. Besides the energy consumption of the electrical components, the in-flight performance of the aircraft is primarily affected by the system’s mass, shape and position. In addition to the flow-induced oscillations, as described in Section 2.2.2, these parameters are elaborated to such an extent that it gives the essentials in design considerations in the context to aircraft performance.

#### 2.6.1. Impact of Weight

Aerial vehicles stay afloat in the air by generating a force that is equal and opposite to the Earth’s gravitational force. In the case of conventional fixed-wing and rotary-wing aircraft, creating such a force requires the consumption of energy. To generate such a thrust force, a rotary-wing aircraft utilizes one or more powered propellers, which are positioned so that it directly counteracts the gravitational force. A fixed-wing aircraft utilizes one or more propellers to generate a forward motion, which results in the main wings to generate a lift force that opposes the gravitational force. As the total mass of the aircraft increases, so does the required lift force. This results in an increase in energy consumption.

In an attempt to demonstrate the importance of weight reduction, the effects of weight on the energy consumption of the aircraft are quantified. For both fixed-wing and rotary-wing sUAS, the maximum range by approximation is reduced proportional to the increase in weight. For sUAS with constant mission variables, the maximum endurance is reduced by a factor of approximately *W*^3/2^, where *W* is the weight, for both fixed-wing [21,38] and rotary-wing [39]. Therefore, an increase of 5% on weight, for example, means a reduction of approximately 7% of endurance.

#### 2.6.2. Impact of Shape and Size

Moving objects placed inside a viscous medium, such as air, are bound to create external forces. Overcoming the effects of such forces typically increases the in-flight power requirements. A stabilized imaging system that is placed inside moving air will contribute to the total drag force and will thus typically reduce the overall in-flight efficiency. It is therefore warranted to optimize the design of the system to reduce the impact through aerodynamic considerations.

#### 2.6.3. Impact of Shape and Size on Fixed-Wing Aircraft

**Figure 12:**
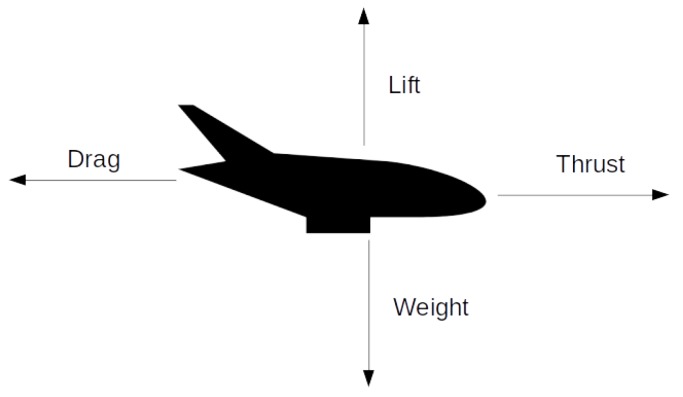
Aerodynamic forces acting on an airplane—thrust, lift and drag.

Aerodynamic drag, also known as air resistance, is the force parallel to the airspeed [40] (Figure 12). With an increase in drag, the aircraft needs to compensate the energy losses by producing more thrust in order not to lose speed or altitude. Considering that a typical stabilized imaging system is not intended to generate a lift force, it may be assumed that the in-flight performance benefits from minimizing the total aerodynamic drag generated by system. To be able to reduce the aerodynamic drag, it is important to understand how it is built up and how it is affected. The total drag force *D* generated by an object can be determined through:

(2) $$D=C_{D}\frac{1}{2}\rho v_{a}^{2}A,$$

where

(3) $$C_{D}={C_{D}}_{P}+{C_{D}}_{I},$$

where *ρ* is the air density in kilogram per cubic meter, *v_a_* is the speed of the moving air before being affected by the object in meters per second, *A* is the cross-sectional area of the object in square meters, and finally *C_D_* is a dimensionless drag coefficient that relates the object’s shape, inclination and flow conditions to the resulting drag force.

**Figure 13:**
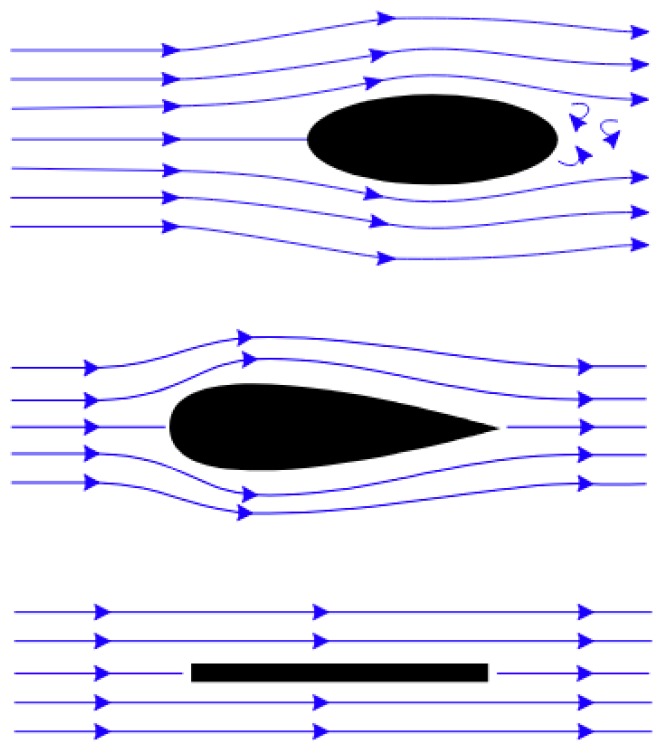
Flow visualization over different shapes (modified from MikeRun/CC-BY-SA-3.0).

The takeaways from Equation (2) in the context of stabilized imaging systems design are that the drag generally can be reduced by minimizing the size of the object, and that the drag increases exponentially with airspeed. Thus, fast flying aircraft suffer much more from poor design choices than slow flying aircraft. Finally, the drag coefficient *C_D_* should be minimized. This can be done by optimizing the shape or placement of the object in such a way that it causes the least interference with the moving air. It should be noted that the complete theoretical basis of aerodynamic flow optimization falls beyond the scope of the study presented here, and is therefore limited to the most relevant aspects. Assuming that the casing of the camera system is not designed to generate lift, the lift-induced drag (${C_{D}}_{I}$) is negligible. The remaining parasitic drag (${C_{D}}_{P}$) can be categorized into:

1. Form drag: The form drag is influenced by the shape of the object (Figure 13). Although the droplet shape offers the most favorable aerodynamic characteristics, when pointed straight into the direction of the moving air, it also offers challenges related to possible viewing angles of the camera system. Therefore, the aerodynamic considerations may be considered a performance parameter within the overall geometrical optimization of the camera system.
2. Skin friction drag: As air moves over the surface of the body, close to the surface the flow will lose energy due to viscous effects. This type of drag is called skin friction drag. A turbulent boundary layer that is induced by a rougher surface may stay attached longer than a laminar boundary layer, thus reducing the form drag. This generally holds true for smaller object in relatively low air speeds [21]. Therefore, for smaller objects, the negative effects of higher skin friction drag, which is caused by a rougher surface, may potentially be offset by a lower overall profile drag. Finally, it should be noted that such potential benefits are highly dependent on the specific design and flow conditions, and therefore require an in-depth aerodynamic analysis. Projects where such analyses is not within reach may benefit from focusing on the form drag and interference drag instead.
3. Interference drag: In the context of aerodynamics, the interference drag can be explained as the airflow over one object disturbing the airflow over another object unfavorably. The actual effects of interference drag depend to a large extent on the airspeed. Therefore, in the context of aircraft performance, it cannot be said that a closed and shielded system is necessarily superior to a system with exposed components as it may also be heavier. It is dependent on mission-specific parameters. The following section suggests methods to study the effects of on-board camera designs, including the interference drag characteristics.

To quantify the actual impact of a camera design on the aircraft performance, there are three methods that should be considered. The first is through wind tunnel testing. Inside a wind tunnel the effects of moving air over an object are recreated, and the generated forces and moments are captured. The camera can be placed inside the wind tunnel without the airframe. However, in that case, the aerodynamic interactions between the aircraft body and stabilized imaging system are not included. If executed and post-processed correctly, a wind tunnel test can give an accurate indication of the impact of the camera system on the flight performance. In addition, the airflow around the bodies can be visualized, which provides information for further optimizing the airflow. However, modeling through wind tunnel experiments is complex and requires dedicated equipment. This causes wind tunnel experiments to be relatively expensive and time consuming. The iterative design process is commonly also slow when compared to its alternatives.

Nowadays, designers have embraced Computational Fluid Dynamics (CFD). This is a computer-based method that can approximate the behaviour of fluids, such as air, over an object. It may prove useful for design optimization as it allows for quicker iterative development. In popular terms, it is sometimes called a virtual wind tunnel. It is to be noted that setting up such a simulation environment requires in-depth knowledge on the theory of fluid dynamics. In addition, when results with a high accuracy are required, a verification of the model is necessary. This is commonly done inside a wind tunnel. This is especially true for low velocities and/or smaller objects, such as camera systems, as the modeling of drag then becomes increasingly problematic.

The final method discussed here is through actual flight tests where the performance is compared with and without the camera system installed. Measuring the consumed energy in cruise flight may serve as an indicative measurement for the impact on the in-flight performance of the aircraft. Such in-flight comparisons are only valid when all mission parameters, including airspeed, altitude, atmospheric conditions and battery charge, are the same in each benchmark flights. Since this may be hard to accomplish and verify, it is important to note that the obtained results are non-conclusive, and can only serve as an approximation. The advantage of this method is that it is accessible and does not require in-depth knowledge of fluid dynamics. This may serve as a suitable method when an approximation is sufficient.

##### Impact of Shape and Size on Rotary-Wing Aircraft

**Figure 14:**
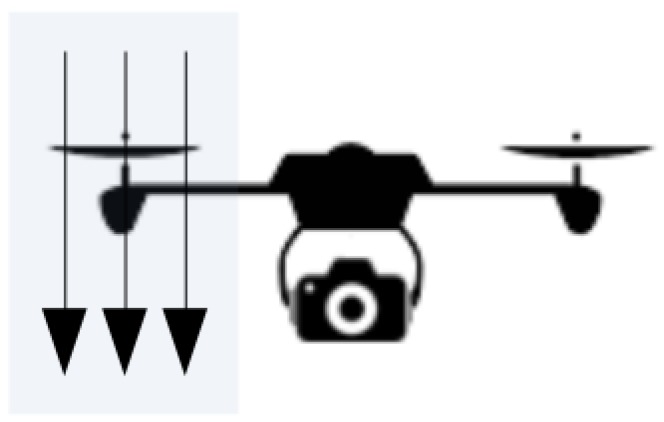
Visualization of the propeller induced slipstream vs in relation to the camera location.

For rotary aircraft operating in stationary flight, there is no forward motion of the vehicle. As the air in front of the rotor is accelerated by the rotors itself, the above-mentioned aerodynamic effects require additional design considerations. By locating the camera system outside of the propeller slipstream (*v_s_*), the impact of the aerodynamic effects are limited (Figure 14). When the aerodynamic effects as a result become small, such a system may not require further aerodynamic optimization. Note, however, that when operating in atmospheric winds the aerodynamic effects remain. For rotary-wing aircraft, a center position should be considered. For conventional rotary aircrafts, which typically utilize one main rotor, the camera mounting points are commonly found under the main body. As the aircraft’s main body shields the camera system to a large extent from the propeller slipstream, the need for aerodynamic optimization decreases. However, due to other design considerations, such as clearance to the ground, these mounting locations may not be feasible. For conventional rotary-wing aircraft, the camera systems are often found to be mechanically suspended in front or along the side of the aircraft body. In such cases, the aircraft performance benefits from a minimized wetted area, which is the area exposed to the airflow, in order to reduce the interference drag and propeller blockage.

#### 2.6.4. Impact of Position

For fixed-wing and rotary-wing aircraft to be controllable in flight, it relies on the ability of the aircraft to compensate the generated forces which are experienced. Each individual component installed on an aircraft has a mass. When these components are exposed to the moving air, it will create a drag force, while lifting bodies may also generate a lift force. First, for the aircraft to maintain altitude in the air, the aircraft needs to be able to generate enough lift force (or thrust) to compensate the aircraft’s total weight. In other words, for level flight the sum of the vertical forces equals zero. In addition, all these individual forces, such as lift and drag, will generate a moment around the aircraft’s center of gravity (C.G.).

## 3. Results and Discussion

Vibration is one of the main concerns when designing sUAS optical imaging systems. In fixed-wing platforms equipped with a combustion engine, the engine is the main source of vibration, followed by the turbulent aerodynamic flow. Rotary-wing platforms have the rotors as the main source of vibration. To mitigate the effects of vibration, mechanical solutions such as dampers can be used. In addition, it is possible to use optical stabilization by installing motion sensors to measure the jitter and use actuators to move the lens in order to correct it. Software solutions are also available, such as digital image stabilization or digital video stabilization algorithms. Gimbal stabilization platforms can be used for stabilization as well as for pointing. The most important components in these platforms are the drive systems and the motion sensors. The installation of a stabilization platform may affect the sUAS aerodynamics and the impact of weight, shape, size and position must be taken into consideration in the design phase.

## 4. Conclusions

In this work, we present a wide survey of the literature about optical imaging stabilization systems and techniques applied to Unmanned Aerial Systems (UAS). This includes discussions about the sources of vibration, how to mitigate its effect using mechanical and software solutions, and the effects of stabilization platforms on the UAS aerodynamics.

## References

[1] Eggleston B., McLuckie B., Koski W.R., Bird D., Patterson C., Bohdanov D., Liu H., Mathews T., Gamage G. (2015). Development of the Brican TD100 Small UAS and Payload Trials. ISPRS Int. Arch. Photogramm. Remote Sens. Spat. Inf. Sci..

[2] Leira F.S., Trnka K., Fossen T.I., Johansen T.A. A ligth-weight thermal camera payload with georeferencing capabilities for small fixed-wing UAVs. Proceedings of the 2015 International Conference on Unmanned Aircraft Systems (ICUAS).

[3] Leira F.S., Johansen T.A., Fossen T.I. Automatic detection, classification and tracking of objects in the ocean surface from UAVs using a thermal camera. Proceedings of the IEEE Aerospace Conference.

[4] Chen J., Wu J., Chen G., Dong W., Sheng X. Design and Development of a Multi-rotor Unmanned Aerial Vehicle System for Bridge Inspection. Proceedings of the Intelligent Robotics and Applications: 9th International Conference (ICIRA 2016).

[5] Shahbazi M., Sohn G., Théau J., Menard P. (2015). Development and Evaluation of a UAV-Photogrammetry System for Precise 3D Environmental Modeling. Sensors.

[6] Hilkert J. (2008). Inertially stabilized platform technology Concepts and principles. IEEE Control Syst. Mag..

[7] Masten M. (2008). Inertially stabilized platforms for optical imaging systems. IEEE Control Syst. Mag..

[8] Li Z., Yan Y., Jing Y., Zhao S. (2015). The Design and Testing of a LiDAR Platform for a UAV for Heritage Mapping. Int. Arch. Photogramm. Remote Sens. Spat. Inf. Sci..

[9] Ma H., Wu J. Analysis of positioning errors caused by platform vibration of airborne LiDAR system. Proceedings of the 2012 8th IEEE International Symposium on Instrumentation and Control Technology (ISICT).

[10] Uragun B., Tansel I.N. The noise reduction techniques for unmanned air vehicles. Proceedings of the 2014 International Conference on Unmanned Aircraft Systems (ICUAS).

[11] Li Z., Lao M., Phang S.K., Hamid M.R.A., Tang K.Z., Lin F. Development and Design Methodology of an Anti-Vibration System on Micro-UAVs. Proceedings of the International Micro Air Vehicle Conference and Flight Competition (IMAV).

[12] Verbeke J., Debruyne S. Vibration analysis of a UAV multirotor frame. Proceedings of the ISMA 2016 International Conference on Noise and Vibration Engineering.

[13] Li C., Tan F. Effect of UAV Vibration on Imaging Quality of Binary Optical Elements. Proceedings of the 2018 IEEE International Conference on Mechatronics and Automation (ICMA).

[14] Gurtner A., Walker R., Boles W. Vibration compensation for fisheye lenses in UAV applications. Proceedings of the 9th Biennial Conference of the Australian Pattern Recognition Society on Digital Image Computing Techniques and Applications.

[15] Gurtner A., Greer D.G., Glassock R., Mejias L., Walker R.A., Boles W.W. (2009). Investigation of fish-eye lenses for small-UAV aerial photography. IEEE Trans. Geosci. Remote Sens..

[16] Haghshenas J. (2015). Effects of satellite platform’s vibrations on the image quality of a remote sensing payload: system level design and challenges. Optical Systems Design 2015: Optical Design and Engineering VI.

[17] Wulich D., Kopeika N. (1987). Image resolution limits resulting from mechanical vibrations. Opt. Eng..

[18] Mizui M., Yamamoto I., Ohsawa R. (2012). Effects of propeller-balance on sensors in small-scale unmanned aerial vehicle. IOSRJEN.

[19] Doty M.J., Fuller C.R., Schiller N.H., Turner T.L. (2013). Active Noise Control of Radiated Noise from Jets.

[20] Yechout T. (2014). Introduction to Aircraft Flight Mechanics.

[21] Raymer D. (2012). Aircraft Design: A Conceptual Approach 5e and RDSWin STUDENT.

[22] Yeom D. (2009). Optical image stabilizer for digital photographing apparatus. IEEE Trans. Consum. Electron..

[23] Li T.H.S., Chen C.C., Su Y.T. (2012). Optical image stabilizing system using fuzzy sliding-mode controller for digital cameras. IEEE Trans. Consum. Electron..

[24] Tico M. (2009). Digital Image Stabilization. Recent Advances in Signal Processing.

[25] Reddy K., Lokesha H., Akhila S. (2015). Video stabilization for micro air vehicles. Int. J. Adv. Res. Comput. Commun. Eng..

[26] Johansen D., Hall J., Beard R., Taylor C. Stabilization of video from miniature air vehicles. Proceedings of the AIAA Guidance, Navigation and Control Conference and Exhibit.

[27] Bosco A., Bruna A., Battiato S., Bella G., Puglisi G. (2008). Digital Video Stabilization through Curve Warping Techniques. IEEE Trans. Consum. Electron..

[28] Thillainayagi R., Kumar K.S. Video stabilization technique for thermal infrared Aerial surveillance. Proceedings of the 2016 Online International Conference on Green Engineering and Technologies (IC-GET).

[29] Shen H., Pan Q., Cheng Y., Yu Y. Fast video stabilization algorithm for UAV. Proceedings of the IEEE International Conference on Intelligent Computing and Intelligent Systems.

[30] Odelga M., Kochanek N., Bülthoff H.H. Efficient real-time video stabilization for UAVs using only IMU data. Proceedings of the 2017 Workshop on Research, Education and Development of Unmanned Aerial Systems (RED-UAS).

[31] Ryu Y.G., Roh H.C., Kim S.J., An K.H., Chung M.J. Digital Image Stabilization for humanoid eyes inspired by human VOR system. Proceedings of the IEEE International Conference on Robotics and Biomimetics (ROBIO).

[32] Albrektsen S., Johansen T. (2018). User-Configurable Timing and Navigation for UAVs. Sensors.

[33] Miller R., Mooty G., Hilkert J.M. (2013). Gimbal system configurations and line-of-sight control techniques for small UAV applications. Airborne Intelligence, Surveillance, Reconnaissance (ISR) Systems and Applications X.

[34] Brake N.J. (2012). Control System Development for Small UAV Gimbal. Master’s Thesis.

[35] Caratao Z.D., Gabel K.F., Arun A., Myers B., Swartzendruber D.L., Lum C.W. MicaSense Aerial Pointing and Stabilization System: Dampening In-Flight Vibrations for Improved Agricultural Imaging. Proceedings of the 2018 AIAA Information Systems-AIAA Infotech@ Aerospace.

[36] Skjong E., Nundal S. (2014). Tracking Objects with Fixed-Wing UAV Using Model Predictive Control and Machine Vision. Master’s Thesis.

[37] Kennedy P.J., Kennedy R.L. (2003). Direct versus indirect line of sight (LOS) stabilization. IEEE Trans. Control Syst. Technol..

[38] Hovenburg A.R., Johansen T.A., Storvold R. Mission performance trade-offs of battery-powered suas. Proceedings of the 2017 International Conference on Unmanned Aircraft Systems (ICUAS).

[39] Leishman G.J. (2006). Principles of Helicopter Aerodynamics.

[40] Gudmundsson S. (2013). General Aviation Aircraft Design: Applied Methods and Procedures.

